# Accurate and Strict Identification of Probiotic Species Based on Coverage of Whole-Metagenome Shotgun Sequencing Data

**DOI:** 10.3389/fmicb.2019.01683

**Published:** 2019-08-07

**Authors:** Donghyeok Seol, So Yun Jhang, Hyaekang Kim, Se-Young Kim, Hyo-Sun Kwak, Soon Han Kim, Woojung Lee, Sewook Park, Heebal Kim, Seoae Cho, Woori Kwak

**Affiliations:** ^1^C&K Genomics, Songpa-gu, South Korea; ^2^Department of Agricultural Biotechnology and Research Institute of Agriculture and Life Sciences, Seoul National University, Seoul, South Korea; ^3^Interdisciplinary Program in Bioinformatics, Seoul National University, Seoul, South Korea; ^4^R&D Center, CTCBIO, Inc., Hwaseong-si, South Korea; ^5^Division of Microbiology, Ministry of Food and Drug Safety, Cheongju-si, South Korea

**Keywords:** NGS, probiotics, lactic acid bacteria, whole genome shotgun sequencing, mapping coverage, identification, metagenomics

## Abstract

Identifying the microbes present in probiotic products is an important issue in product quality control and public health. The most common methods used to identify genera containing species that produce lactic acid are matrix-assisted laser desorption/ionization–time of flight mass spectrometry (MALDI-TOF MS) and 16S rRNA sequence analysis. However, the high cost of operation, difficulty in distinguishing between similar species, and limitations of the current sequencing technologies have made it difficult to obtain accurate results using these tools. To overcome these problems, a whole-genome shotgun sequencing approach has been developed along with various metagenomic classification tools. Widely used tools include the marker gene and *k*-mer methods, but their inevitable false-positives (FPs) hampered an accurate analysis. We therefore, designed a coverage-based pipeline to reduce the FP problem and to achieve a more reliable identification of species. The coverage-based pipeline described here not only shows higher accuracy for the detection of species and proportion analysis, based on mapping depth, but can be applied regardless of the sequencing platform. We believe that the coverage-based pipeline described in this study can provide appropriate support for probiotic quality control, addressing current labeling issues.

## Introduction

In light of the trend toward increasing interest in health, many probiotic products are emerging. The global probiotics market exceeded 40 billion USD in 2017 and more than 12 million tons of these products are expected to be consumed by 2024^1^. Probiotics are now used not only for nutrition, but also for medical purposes, such as to promote the development of the infant immune system (O’Toole et al., 2017; Michelini et al., 2018). In this growing market, defective products are also increasing, which can pose some risks to consumers (Lewis et al., 2015). Although authorities such as the United States Food and Drug Administration (FDA) check all probiotic products before they permit their sale, they could pass products without knowing whether the bacteria in these products might be mislabeled. Thus, to safely manage the probiotic market, it is necessary to verify whether probiotic products actually contain the species mentioned on their labels. Such genera include *Lactobacillus*, *Bifidobacterium*, and *Bacillus*, which are referred to as genera containing species that produce lactic acid (GSLA) throughout this manuscript.

There are many ways to identify GSLA at the species level (Herbel et al., 2013), such as matrix-assisted laser desorption/ionization–time of flight mass spectrometry (MALDI-TOF MS) and 16S rRNA sequence analysis (Angelakis et al., 2011; Garcia et al., 2016). For MALDI-TOF MS, the initial cost is high (Wieser et al., 2012) and the approach to identifying species is library-based, which may lead to difficulty detecting species that are not listed in the spectral database (Singhal et al., 2015). Even if information is present in the database, being able to accurately identify similar species remains a challenge (Dušková et al., 2012; Bailey et al., 2013). In a similar manner, 16S rRNA sequences may be difficult to analyze because full-length 16S rRNA must be read for accurate profiling, and the sequencing must be carried out with high accuracy (Edgar, 2018a,b). Notably, the Illumina and Ion Torrent platforms are based on short read lengths of less than 400 bp (Hodkinson and Grice, 2014) which makes it difficult to compare 1,600 bp, the full length of the 16S rRNA gene, with sequences in public databases (Yang et al., 2011). Conversely, the Pacbio and Nanopore platforms are capable of long read sequencing over 2,000 bp, but with error rates of more than 10% (Rhoads and Au, 2015); thus, comparison of 16S rRNA at the 97% similarity level for species classification is not suitable (Wagner et al., 2016). Although the circular consensus sequencing (CCS) method of Pacbio can read the full length of 16S rRNA with high accuracy (Frank et al., 2016; Pootakham et al., 2017), it costs more than the common 16S amplicon method used by the Illumina platform.

As a solution to the above problems, the whole genome shotgun sequencing method has been proposed and widely applied in numerous microbial community analyses (Loman et al., 2012; Quince et al., 2017). One requirement for the whole-genome shotgun sequencing approach is metagenomic classification, which can follow various strategies (Breitwieser et al., 2017) including matching k-mers [e.g., Kraken (Wood and Salzberg, 2014), k-SLAM (Ainsworth et al., 2017), and CLARK (Ounit et al., 2015)], aligning to marker genes [e.g., MetaPhlAn 2 (Truong et al., 2015) and GOTTCHA (Freitas et al., 2015)] and translating into amino acid sequences [e.g., Kaiju (Menzel et al., 2016)]. These methods use a specific region of interest for detection instead of the whole genome, causing markers to lose their specificity. For example, if a new species is not available as a reference due to the absence of assembly data, but shares similar regions with other species due to horizontal gene transfer (HGT) (Hiraoka et al., 2016), the markers may detect other species. In addition, sequencing and assembly errors in the reference data can affect the detection of species, causing problems if it is necessary to rigorously determine the presence or absence of a species (Peabody et al., 2015).

In this study, we introduce a new GSLA classification pipeline that effectively reduces the false-positive (FP) rate using mapping coverage. The coverage yielded by alignment to the representative strain of a species was the coverage criterion. Due to the fact that the classification pipeline was based on the whole genome, the accuracy of the proportion analysis based on mapping depth was high, and FPs at the species level were not present; thus, more reliable results were achieved than with other metagenomic classification methods. We expect that the coverage-based pipeline presented in this study will facilitate efficient quality control of probiotic products, as well as the relabeling of products with inaccurate information. Overall, application of our pipeline could have a positive contribution to public health.

## Materials and Methods

**FIGURE 1:**
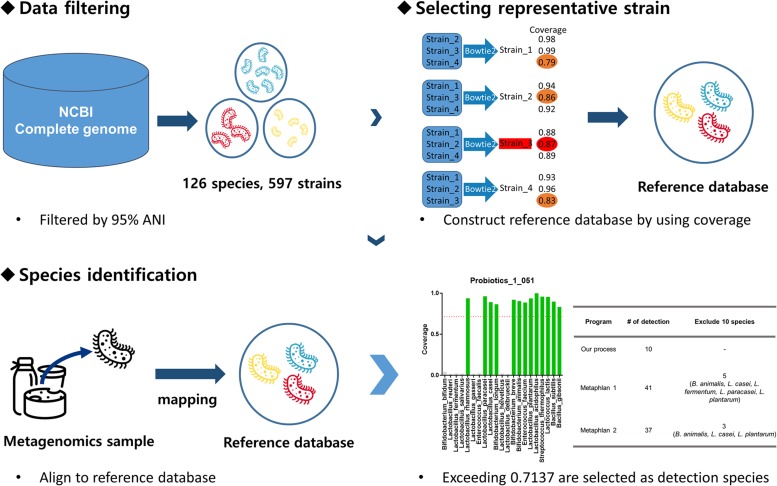
Pipeline overview. First, the complete genome data set was downloaded from the NCBI and filtered based on 95% ANI to obtain 126 species and 597 strains. Then, the one-to-one pairwise coverage of all strains of each species was calculated. In this case, the strain with the highest minimum coverage value for a given species was selected as the representative strain for that species. For example, if there are four strains of a species, we consider each of the strains as a reference strain; the coverage is calculated by aligning sequence data of the remaining three strains to the reference strain using bowtie2. Afterward, the minimum coverage of each strain is compared (Orange: strain_1: 0.79, strain_2: 0.86, strain_3: 0.87, strain_4: 0.83) and that with the highest value is selected (Red: strain_3: 0.87). Thus, the strain with the highest coverage is the representative strain of that species. A reference database was constructed using the representative strains and whole-metagenome shotgun sequencing data of probiotic probiotics were aligned to it. Only species exceeding 0.7137 coverage were judged to be present in the probiotic product.

Our pipeline consists of two stages: database construction and species detection. During the database construction stage, one-to-one coverage was calculated for each species of GSLA, and a representative strain was selected for construction of a database to detect that species. Based on coverage, the detection threshold was also determined. During the second stage, the probiotic metagenomic data were mapped to the database created in the first stage. Species exceeding the coverage threshold were recorded as the detected species. A more detailed explanation of the GSLA detection pipeline is provided in Figure 1.

### Determination of the Representative Strain

The complete genomes of 126 species and 597 strains of GSLA were downloaded from the National Center for Biotechnology Information (NCBI^2^) (Supplementary Table S1). One-to-one pairs of average nucleotide identity (ANI) were obtained within species and filtered at a threshold of 95% identity. Illumina paired-end simulated data were generated using the ART simulator (art_illumina) program with the following parameters, based on the HiSeq 2000 platform (2 × 100 bp): mean size of DNA fragments: 350 bp, read coverage: 100 fold, and standard deviation of DNA fragment size: 10 (Huang et al., 2012). The reference genome was assigned one-to-one in the manner described above to determine coverage using bowtie2 with default settings (Langmead and Salzberg, 2012). After comparing the minimum coverage value by setting different strains as the reference genome, the strain with the highest minimum coverage value was selected as the representative strain for that species. At this point, if subspecies existed within a given species, if any strain group had an ANI value less than 95%, despite belonging to the same species, or if more than two groups clustered distinctly on the heatmap of all pairwise one-to-one ANI values, we selected additional representative strains. After that, the coverage threshold for detecting GSLA species was set to the lowest minimum coverage value out of the representative strain selected for each species. A reference set was then constructed for GSLA classification by combining the representative strains into a multi-FASTA file. In order to determine the coverage criterion, the values obtained from mapping the sequence reads to only one representative strain and to all representative strains combined into a set must be similar. This is because it explains how accurately the sequence reads are aligned to the representative strain of the species to which they belong.

### Sequencing of Probiotic Products

We sequenced the GSLA species in six probiotic products: one with Illumina and five with Ion Torrent technology. Considering that the Illumina platform was used for processing the simulated data and data from the NCBI sequence read archive (SRA^3^), and produced reliable results, testing real data using a different sequencing platform, such as the Ion Torrent, can reduce the platform bias of our pipeline. With the Illumina platform, library preparation was carried out using the TruSeq Nano DNA LT Kit (Illumina), and sequencing was then conducted using the NextSeq 500 sequencer (Illumina) in paired-end read mode. The read length was 150 bp per read. With the Ion Torrent platform, the prepared libraries were sequenced using the Ion S5 sequencer (Ion Torrent) and the read length was 350 bp.

### Detection Ability Test

Whole-genome shotgun sequencing data for a single species were downloaded from NCBI SRA and mapped to a reference set to determine whether that bacterial species was present. If two or more bacteria were detected that could not be distinguished based on the ANI criterion, an additional analysis was conducted. In this additional analysis, all complete genomes of the species identified in the detection test were used as reference sequences and aligned using the bowtie2 options of “–a (search for all alignments)” and “–a –score-min ‘C,0,–1’ (search for all alignments with perfect match).” The species with the highest resulting coverage was designated as the detected species.

Next, to examine the detection capability of the metagenomics method, the program performed three processing steps to yield simulated data, SRA data, and real probiotic sample data. First, using simulated data, we created one large metagenome dataset by combining reads for 13 species obtained through ART simulation (Supplementary Table S2). Second, data for 10 different species obtained from SRA, which were all collected with the same platform and read length, were downloaded from NCBI and combined into one dataset (Supplementary Table S3). Finally, to examine the detection capability of bacteria in actual probiotics using whole-genome shotgun sequencing data, we used Illumina paired-end read data for 19 GSLA, and Ion Torrent platform data for 4∼11 GSLA. We used Trimmomatic (TRAILING: 30) for quality control. For Ion Torrent platform data, we used the TMAP aligner instead of bowtie2 as an alignment program, with the setting of stage 1 map 4. For the 30 Gb ^*^ 2 of Illumina data, the processing time required was measured with the file size reduced to 15 Gb ^*^ 2, 7.5 Gb ^*^ 2, 3 Gb ^*^ 2, and 1.5 Gb ^*^ 2 through random sampling.

Subsequently, the complete genomes of 19 GSLA species approved by the Ministry of Food and Drug Safety (MFDS; Korean Food & Drug Administration) as probiotics were used to calculate the proportional abundance of the species in the sample (Supplementary Table S4). All the reported strains of 19 species at the complete genome level were concatenated according to species, to create a single FASTA file. A reference dataset was then constructed for proportion analysis by combining all of these files into a multi-FASTA file. The species proportions were calculated according to the relative ratio of the mapping depth of a given group, divided by the average length of sequences for that group. Furthermore, only simulated and SRA data were used, and we have combined 10 species to have equal proportions of 10%. For simulated data, we used a number of reads for each species that was in proportion to the sequence length for that species, to simulate the actual data product (Supplementary Table S5). For SRA data, we carried out additional analysis to identify the most similar strain to each downloaded SRA sequence, and the read count in proportion to the sequence length of that strain, and then combined these strains into one dataset. We also used the same data as for the detection capability test. All of these detectability tests were repeated using several other metagenomic classifiers, such as MetaPhlAn 1 (Segata et al., 2012), MetaPhlAn 2 (Truong et al., 2015), Kaiju (Menzel et al., 2016), k-SLAM (Ainsworth et al., 2017), CLARK-S (Ounit and Lonardi, 2016), and KrakenHLL (Breitwieser and Salzberg, 2018) for a comparison of the results with those from our pipeline.

### Data Availability

The sequencing data analyzed for this study are available via the NCBI Sequence Read Archive (SRA) under accession number BioProject PRJNA508569. The document for python source code and the reference sequence data index file used for detection and proportion analysis in this study are freely available from the Github repository^4^ and Google Drive^5^.

## Results

### Building a Representative Genome Set and Determining the Coverage Criterion

In this study, complete genomes for a total of 126 species and 597 strains of GSLA were downloaded from NCBI (Supplementary Table S1). Rather than using all 597 strains, we selected representative strains for each species to form a representative genome set, due to high sequence identity among the genomes of strains within a species. The representative strain was that having the highest minimum coverage in all pairwise comparisons between genomes of strains within a given species. Before analyzing the coverage data, ANI analysis was performed to verify whether the genomes represented the same species. In general, if ANI exceeds 95%, genomes can be classified as the same species (Goris et al., 2007). However, pairwise ANI calculations showed that some strain genomes did not exceed the ANI criterion despite being from the same species. In our research, *Bacillus pumilus*, *Bacillus amyloliquefaciens*, *Lactobacillus casei*, and *Lactococcus lactis* contained strains that were not considered to be of the same species, and which were instead classified into two groups based on 95% ANI (Supplementary Figure S1). For example, when mapping a shotgun read simulated from the genome of the *L. casei* type strain (GCF_000019245.4) to a genome of *L. casei* (GCF_000829055.1) in another group, we found that the read mapping coverage was very low, to the extent that it cannot be regarded as the same species (Supplementary Figure S2; Fontana et al., 2018). Although *L. casei* did not have any officially named subspecies, the nine strains analyzed were divided in two groups consisting of seven and two strains, with the latter two strains being *L. casei* LC5 and *L. casei* ATCC 393. Similarly, *B. pumilus* had no officially named subspecies, but it was consistent with the previous work in which the whole genome phylogenetic tree analysis showed that *B. pumilus* was divided into two clades. One of the clades was clustered with *Bacillus altitudinis* (Tirumalai et al., 2018). Moreover, *L. lactis* was classified into two groups based on 95% ANI, such that it explained the presence of two subspecies, *L. lactis* subsp. *lactis* and *L. lactis* subsp. *cremoris*, through their NCBI accession numbers (Salama et al., 1991). Another study also showed the presence of two subspecies for *B. amyloliquefaciens*, which were *B. amyloliquefaciens* subsp. *amyloliquefaciens* and *B. amyloliquefaciens* subsp. *plantarum* along with the result of the ANI analysis (Borriss et al., 2011). Therefore, the classification of two groups indicated the subspecies within those species.

Unlike the species listed above, *Bifidobacterium longum* is reported to have three subspecies based on the different ANI criterion (Mattarelli et al., 2008). Interestingly, 95% of the ANI cutoff defined *B. longum* as one species, however, it was classified into three subspecies when the cutoff increased to 97% (Supplementary Figure S1E). In order to investigate whether *B. longum* should be divided into three subspecies based on ANI criteria for accurate subspecies classification, we first checked the NCBI accession number of each strain and confirmed that one of the three subspecies was *B. longum* subsp. *infantis*. The other two groups could not be identified based on the NCBI accession number, we therefore indirectly determined whether the subspecies were represented by using data of subspecies of *B. longum* downloaded from the SRA. As a result, the strains were divided into three groups according to the coverage standard: *B. longum* subsp. *longum*, *B. longum* subsp. *suis*, and *B. longum* subsp. *infantis* (Supplementary Figure S3; Mattarelli et al., 2008). Therefore, a total of 132 strains, including three strains of *B. longum*, two strains each of *L. lactis*, *B. pumilus*, *B. amyloliquefaciens*, and *L. casei*, and 121 strains of other individual species were selected for the 126 species analyzed, and a representative genome set was constructed from these sequences (Supplementary Table S6).

**FIGURE 2:**
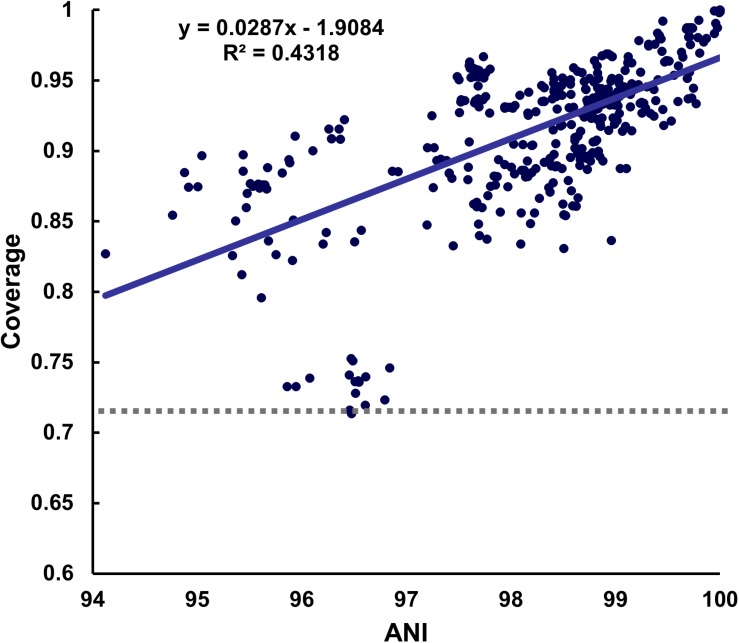
Distribution of coverage values. A positive correlation between the ANI and coverage was observed when the representative strain of each species was used as the reference sequence. The lowest minimum coverage value was 0.7137 at 96.47% ANI in *B. longum*.

In the meantime, when selecting the representative strain, the minimum coverage varied greatly depending on which strain was used. In the case of *B. longum*, for which 18 strains were reported, the minimum coverage was 0.7137 when the representative genome was used, while it reduced to 0.5534 when a non-representative genome from strain GCF_000020425.1 was used (Supplementary Figure S4). In addition, the minimum coverage of 0.7137 was similar to the result of 70% obtained from DNA-DNA hybridization (DDH), which was used for experimental identification (Goris et al., 2007). Furthermore, the highest minimum coverage values for the representative strains ranged between 0.7137 and 0.993 across species (Figure 2). Although the minimum mapping coverage of *B. longum* obtained 0.7137, it increased to 0.8453 when representative strains from each subspecies were considered. However, because the value of 0.8453 was obtained without considering variants of other species that may or may not be present in the reference dataset, we set the lowest value obtained for mapping coverage of all GSLA of 0.7137 as the baseline for species detection.

As we calculated the ANI and mapping coverage, we wanted to see the relationship between them. As a result, it showed a positive correlation in most species, but the strength of this correlation differed among species. For example, the coverage and ANI values for *Enterococcus faecalis* and *Pediococcus pentosaceus* were not related (Supplementary Figure S5).

Meanwhile, the baseline for species detection was assigned when reads were aligned to a single genome. The representative genome set contained 132 strains in total, but the results of read mapping coverage targeting a single genome could differ due to the presence of homologous regions between species. Thus, we checked whether the same results were obtained using only the representative strain versus the entire set of representative genomes as a mapping target. In this test, we used simulated reads of nine strains for two species, *Lactobacillus helveticus* and *Lactobacillus brevis*. No significant difference in mapping coverage was observed (<0.0017) on aligning each strain to the representative strain of the same species, or to the reference set containing all 132 strains (Supplementary Table S7).

### No False Positive Results in Detection Ability Test

We performed a detection test to determine whether the representative genome set, and the baseline were applicable to actual data rather than simulated data. In the detection ability test, four types of data were used. First, single-species data downloaded from NCBI SRA were tested and we then executed the program with data representing various GSLA species in the order of simulated data, SRA, and real data. For single-species data, we investigated the 19 probiotic GSLA species approved by the MFDS; from the SRA data of the 16 species, only one species was correctly detected for each dataset. The maximum coverage of species other than the detected species was as low as 0.01–0.25, confirming that only one species was detected without considering the possibility of false-negative (FN) results. In contrast, two species were identified in SRA data of the following three species: *L. casei*, *Lactobacillus paracasei*, and *L. helveticus* (Supplementary Figure S6). The additional species detected in their data were *L. paracasei*, *L. casei*, and *Lactobacillus gallinarum*, respectively, which were considered as the same species based on the ANI criteria for each species. In the case of *L. casei*, reads comprising the dataset were generated from sequencing only a single strain of *L. casei*. Nonetheless, the sequences of *L. paracasei* and *L. casei* shared similar regions that happened to be aligned in *L. paracasei*, eventually exceeding our mapping coverage baseline for both species. As a result, of the 126 species analyzed in total, seven one-to-one pairs included different species that were classified as same species based on ANI (Table 1). To address this problem, an additional analysis was conducted using the complete genomes of all species that are not distinguishable from other species based on ANI as a reference genome set (i.e., *L. casei* – *L. paracasei* and *L. helveticus* – *L. gallinarum*). Reads were next mapped in all regions using the “–a” option of the bowtie2 program, which is a tool used for aligning all reads at the same loci. Among these reads, the strain with the highest coverage, i.e., that which is most similar to the genome from which the reads were generated, was assigned as the detected species. As a result, all three species were accurately detected: *L. paracasei* with a coverage value of 0.9119, *L. helveticus* with 1 and *L. casei* with 0.9122 (Supplementary Table S8). Because this additional analysis was used to determine all alignments, the time required can vary greatly depending on the size of the reference dataset. In the case described above, it took about 50 min for 600 Mb ^*^ 2 *L. paracasei* Illumina sequencing data to be aligned to the reference genome set containing 18 strains of *L. casei* + *L. paracasei*.

**TABLE 1:** ANI values for closely related pairs of GSLA species.

**Genus**	**Species**	**Species**	**ANI**
*Bacillus*	*B. gibsonii*	*B. subtilis*	98.87
	*B. vallismortis*	*B. velezensis*	98.21
*Bifidobacterium*	*B. catenulatum*	*B. kashiwanohense*	96.56
	*B. coryneforme*	*B. indicum*	98.28
*Lactobacillus*	*L. casei*	*L. paracasei*	97.22
	*L. gallinarum*	*L. helveticus*	98.39
*Leuconostoc*	*L. garlicum*	*L. lactis*	97.93

*ANI, average nucleotide identity.*

Testing our SRA data with MetaPhlAn 1, MetaPhlAn 2, CLARK-S, k-SLAM, Kaiju, and KrakenHLL resulted in multiple FPs, despite the use of single-species data. Seven and nine FPs were obtained from MetaPhlAn 2 and MetaPhlAn 1, respectively. Moreover, several hundred FPs occurred among CLARK-S, k-SLAM, and Kaiju. KrakenHLL provided an ideal threshold for the unique *k*-mer count per sample read (unique *k*−mer = 2000^*^million  read), but up to 11 FPs were still found in the filtered results (Table 2).

**TABLE 2:** The results of single species data from the SRA.

**Species**	**Accession**	**Our process**	**MetaPhlAn 1**	**MetaPhlAn 2**	**CLARK-S**	**k-SLAM**	**Kaiju**	**KrakenHLL**
*L. lactis*	ERX231530	1	1 + 3	1 + 3	1 + 294	1 + 639	1 + 991	1 + 9
*S. thermophilus*	SRX2610845	1	1	1	1 + 122	1 + 54	1 + 207	1 + 5
*L. acidophilus*	SRX2610831	1	1	1	1 + 89	1 + 38	1 + 160	1 + 2
*L. plantarum*	ERX1625346	1	1 + 1	1 + 2	1 + 245	1 + 62	1 + 291	1 + 2
*E. faecium*	ERX2085159	1	1	1	1 + 149	1 + 86	1 + 809	1 + 2
*B. longum*	ERX1960389	1	1 + 1	1	1 + 258	1 + 149	1 + 626	1 + 1
*B. animalis*	SRX2610848	1	1	1	1 + 109	1 + 28	1 + 314	1 + 5
*B. breve*	SRX2610844	1	1 + 2	1	1 + 39	1 + 22	1 + 108	1 + 6
*L. delbrueckii*	ERX231531	1	1 + 4	1 + 4	1 + 346	1 + 257	1 + 1193	1 + 11
*E. faecalis*	ERX2102726	1	1 + 1	1 + 1	1 + 65	1 + 44	1 + 258	1 + 1
*L. rhamnosus*	SRX2610827	1	1 + 2	1 + 1	1 + 53	1 + 31	1 + 87	1 + 5
*L. salivarius*	SRX2268576	1	1 + 1	1 + 2	1 + 169	1 + 83	1 + 353	1 + 7
*L. gasseri*	ERX980028	1	1 + 2	1 + 1	1 + 112	1 + 74	1 + 251	1 + 3
*L. reuteri*	SRX2268579	1	1 + 1	1 + 1	1 + 91	1 + 34	1 + 358	1 + 1
*L. fermentum*	SRX2268582	1	1 + 1	1 + 1	1 + 79	1 + 32	1 + 206	1 + 4
*B. bifidum*	ERX1101269	1	1	1	1 + 125	1 + 77	1 + 569	1 + 3
*L. casei*	SRX1433289	1	1 + 1	1	1 + 276	1 + 96	1 + 490	1 + 3
*L. paracasei*	ERX178725	1	1 + 9	1 + 7	1 + 281	1 + 122	1 + 575	1 + 10
*L. helveticus*	SRX2268585	1	1 + 1	1 + 3	1 + 184	1 + 53	1 + 206	1 + 7

*The number of FP species is represented by “+.”*

Detection ability tests for single species did not allow detection of FNs or FPs, and thus showed perfect results. Nonetheless, if the data are complex due to a mixture of different species, high-identity problems may occur, such as increased coverage of species that are not included in the sample and increased FPs. Moreover, FNs may occur if the sample does not have sufficient coverage of a species that makes up a small proportion of the sample. Therefore, we processed simulated data, SRA, and real data to determine how accurately our pipeline detected species in complex data.

**FIGURE 3:**
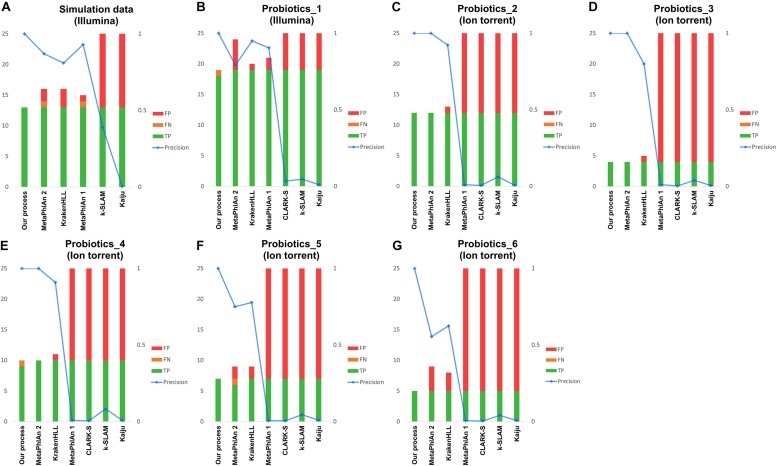
Results of metagenomic analysis of probiotic products. Panel **(A)** shows simulated data, **(B)** shows the real data obtained with the Illumina platform and **(C–G)** show real data from the Ion Torrent platform. Green indicates the number of species correctly detected, yellow indicates FNs, and red indicates FPs. The blue line represents the precision of each classification.

First, the simulated data of 13 species combined using the ART simulator revealed 13 species in our pipeline, but all classifications contained FPs. The numbers of FPs obtained using MetaPhlAn 1, MetaPhlAn 2, KrakenHLL, k-SLAM, and Kaiju were 1, 2, 3, 20, and 2,847, respectively (Figure 3A). Despite the use of simulated data, one FN was found in each of the MetaPhlAn 1 and MetaPhlAn 2 results. Meanwhile, 100% *Campylobacter curvus* was detected using CLARK-S for unknown reasons.

Second, based on the analysis of data for 10 species combined, our pipeline detected 10 species and demonstrated better results than other programs such as MetaPhlAn 1, MetaPhlAn 2 and KrakenHLL, which detected 41, 37, and 32 species, respectively. The other programs using *k*-mers or protein sequence data detected a much greater number of species.

Lastly, real data were analyzed using 19 species in Illumina data, and four to 11 species in Ion Torrent data. In the Illumina data, our pipeline detected 18 species and a FN, whereas MetaPhlAn 1, MetaPhlAn 2, and KrakenHLL detected 19 species along with two, five, and one additional species, respectively (Figure 3B). Among the five Ion Torrent samples analyzed (Figures 3C–G), our pipeline yielded one FN in the Probiotics_4 sample (Figure 3E). In MetaPhlAn 2, false detection occurred in the Probiotics_5 and Probiotics_6 samples; one FN species and two FPs were detected in Probiotics_5 (Figure 3F), and four FP species in Probiotics_6 (Figure 3G). Despite filtering based on the suggested criteria, KrakenHLL resulted FPs across all five probiotic products, with one, one, one, two, and three FPs detected, respectively (Figures 3C–G). MetaPhlAn 1 showed similar performance to MetaPhlAn 2 and KrakenHLL based on data collected on the Illumina platform, but at least 300, and sometimes more than 1,000, FPs were obtained with the Ion Torrent data. CLARK-S, k-SLAM, and Kaiju exhibited more than 100 FPs in all of the tests described above, regardless of platform (Figures 3B–G).

### High Accuracy of Proportion Analysis

**FIGURE 4:**
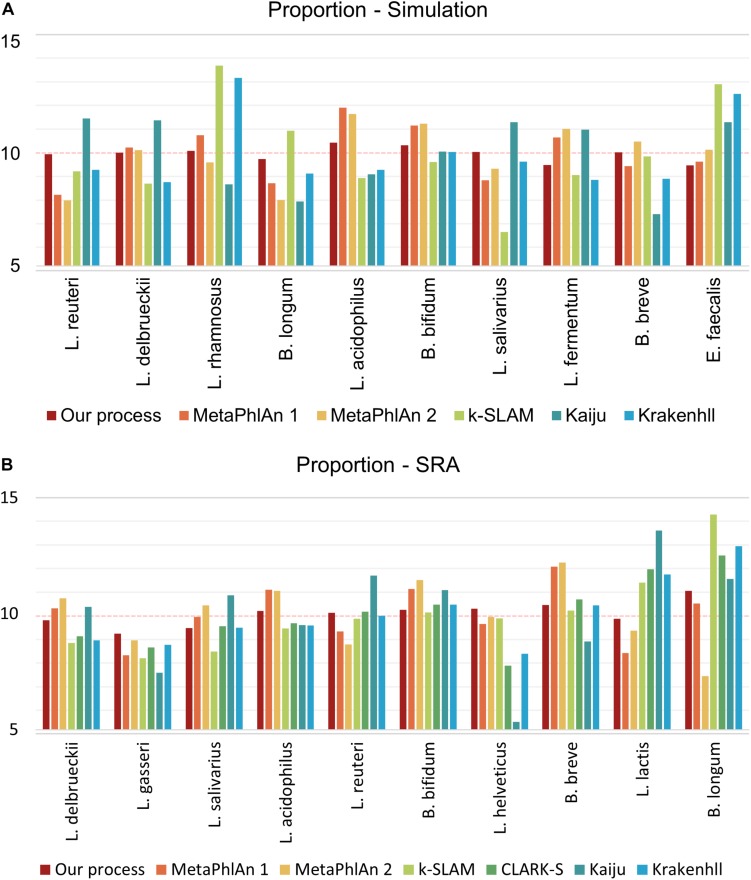
Species proportion analysis of simulated and SRA data. The number of reads was determined in proportion to the genome size of each species, and a total of 10 species were combined. Panel **(A)** shows simulated data, **(B)** shows data from the NCBI SRA. The closer the classification value of each species is to 10, the more accurately the proportions are determined.

To control the quality of probiotic products, it is essential not only to detect the presence of species, but also their relative ratios. The cost of probiotic products varies based on the species present, and species that make up a small proportion of the total bacteria may gradually disappear from a product over time. For proportion analysis, the number of reads as a proportion of the genome size of each species was standardized so that the data showed the same ratio (i.e., 10%) for all 10 species of GSLA. As in the detection ability test described above, FP species appeared in all programs tested except for our pipeline, however, only the relative quantities of the 10 species of interest were compared, without consideration of the FP species. All other programs were executed based on the proportions revealed by their results, whereas the calculation was based on mapping depth for our pipeline. Using simulated data, the variance in proportions was 0.11 in our pipeline, versus 1.56 in MetaPhlAn 1, 1.75 in MetaPhlAn 2, 4.78 in k-SLAM, 2.72 in Kaiju and 2.76 in KrakenHLL (Figure 4A). As in the detection ability test, CLARK-S detected 100% *C. curvus* species. Using SRA data, the variance in proportions was 0.27 for our pipeline, 1.49 for MetaPhlAn 1, 2.15 for MetaPhlAn 2, 3.17 for k-SLAM, 2.12 for CLARK-S, 5.51 for Kaiju and 2.04 for KrakenHLL (Figure 4B).

### Time Required for Species Detection

It is important to determine the number of reads and time required to detect the species when using any method because both time and monetary costs depend on the size of the dataset. Through random sampling, we controlled costs by reducing the size of the Probiotics_1 dataset, which was the largest dataset (30 Gb ^*^ 2) used in the detection ability test.

**FIGURE 5:**
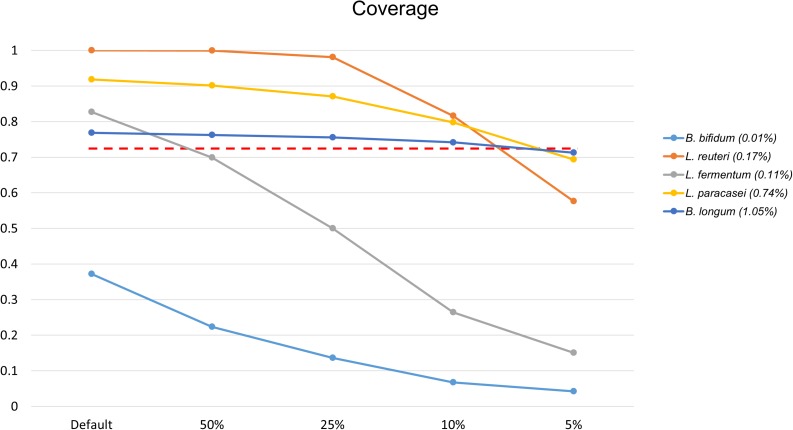
Changes in coverage values for species when reducing the Illumina data file size, of 30 Gb ^*^ 2, to 50, 25, 10, and 5%. Only species that changed to FN results are shown. *L. fermentum* was not detected when the data file size was reduced by 50% (30 Gb ^*^ 2 → 15 Gb ^*^ 2) and *B. longum*, *L. paracasei*, *L. reuteri* were not detected with a reduction to 5% (3 Gb ^*^ 2 → 1.5 Gb ^*^ 2). The red dotted line shows 0.7137 coverage. The number in parentheses in the legend is the proportion analysis result.

*Bifidobacterium bifidum* was not detected at first, while *Lactobacillus fermentum*, with 0.6989 coverage, was not detected when the file size was reduced to 50%. As a result of a further reduction in file size, from 10 to 5%, three additional FNs appeared. At 5% of the original data set size, *B. longum*, *L. paracasei*, and *Lactobacillus reuteri* were not detected, with 0.713, 0.6936, and 0.5766 coverage, respectively (Figure 5). According to proportion analysis of these Illumina data, we confirmed that *B. bifidum* accounted for 0.01% of the sample, *L. fermentum* for 0.11%, *B. longum* for 1.05%, *L. paracasei* for 0.74%, and *L. reuteri* for 0.17%. Considering these results, we determined that at least 3 Gb ^*^ 2 of data was required for species detection in Illumina paired-end data, accounting for about 1% of the sample. When the file size was reduced, the time required for processing was also dramatically reduced: 452 min for 30 Gb ^*^ 2 and 25 min for 3 Gb ^*^ 2 (Table 3).

**TABLE 3:** Data processing time required when the data set size was reduced (Min).

**Method**	**Program (ver.)**	**Default**	**50%**	**25%**	**10%**	**5%**
Alignment	Bowtie 2 (2.3.3.1)	383	105	60	20	10
BAM file sorting	SAMtools (1.3.1)	40	15	8	3	2
Genomecov	Bedtools (2.20.1)	28	10	5	2	1
	Sum	451	130	73	25	13

## Discussion

Our pipeline, which is based on mapping coverage, provides new criteria for determining the presence or absence of GSLA in a sample, adequately controlling for false detections and showing high accuracy in proportion analysis.

A benefit of using all available genome information is that it is possible to address problems such as structural variations in the genome of an individual species. However, when the same loci are present at the mapping target, due to homology, most short-read aligners are randomly mapped to one of them, affecting the calculation of genome coverage for each species. Those reads can be mapped to the same loci by adjusting the alignment parameters, but increases mapping time significantly and adds reads artificially, leading to incorrect results in subsequent proportion analysis. Moreover, due to the difference among strains in the number of genomes available in the current database, it is difficult to set the coverage criterion for the detection test. According to the detection test, there was no difference in performance between the pipeline that used only the genomes of representative strains and that using all available genomes of all strains. Thus, to obtain data for proportion analysis in a shorter time without compromising detection ability, we utilized the representative genome set as reference data.

As this methodology uses only one representative genome for each species, there is a tremendous difference in the results depending on which strain’s genome is used as the representative genome. For example, B. longum had different minimum coverage when the representative genome or a non-representative genome from strain GCF_000020425.1 was used. When the representative genome was used, the coverage was similar to the results of 70% DDH, whereas, when the non-representative genome was used, the coverage reduced so that it was too low to be used as a criterion for mapping coverage. The criterion cannot be too high or too low because of the ability of detection. If it is too low, even species that should not be detected will be detected. Thus, it has to be reasonable, such that the value that was similar to the result of 70% DDH was set as the coverage criterion for our pipeline. Additionally, this result confirms the importance of selecting a representative genome for species determination using our pipeline, as well as, showing why we selected a representative genome for each species by calculating all pairwise minimum coverage values for all strains with available genomic data. The highest minimum coverage values for the representative strains varies across species. This results may have been caused by the myriad of genomic structural variants present in certain species (Lan and Reeves, 2000). For instance, the minimum one-to-one pairwise coverage value for *B. longum* increased when all representative strains were used because it considers the structural variation, compared to when aligning to only one representative strain.

During the process of species identification, two problems were observed: (1) strains that came from the same species separated into different species based on ANI criteria, and (2) two different species grouped together and classified as the same species. The first problem was solved by selecting an additional representative strain for each group that was divided based on 95% ANI. As a result, we were able to identify the strains at the species level regardless of which group they belonged to. However, the other metagenomic classification tools such as MetaPhlAn showed a downside in classifying species. For example, in the detection ability test, most samples with *L. casei* had high coverage, with the representative strain of the group containing seven strains. Meanwhile, in two samples, i.e., the simulated data and Probiotics_5 on Ion Torrent, the coverage for the representative strain of the two strain groups, and not that for the seven-strain group, was greater than 0.7137. In these samples, MetaPhlAn showed false detections, while MetaPhlAn 1 did not detect *L. casei* at all and MetaPhlAn 2 detected *Lactobacillus zeae* as a FP instead of *L. casei*. This FP occurred because *L. zeae* falls under the *L. casei* group based on NCBI taxonomy, and the two strains were very similar to *L. zeae* (Kang et al., 2017). For the second case, the problem is that two species were detected even though the sample contains only one species. For example, *L. gallinarum* was detected in *L. helveticus* single-species data, because these species have high identity (Jebava et al., 2014). In other words, the two species shared reads used in the process of aligning. To prevent this issue, it was necessary to accurately classify the data through additional analysis. However, if the proportion of that GSLA in the product was low or if insufficient sequencing data were produced, both species may be undetected due to their shared reads. Therefore, in such situations, only one species per pair was included in the reference set to ensure sufficient coverage, and when that species was detected, accurate species detection was carried out through additional analysis. However, whether or not both species in a pair are present in a product remains to be addressed. It is therefore necessary to reclassify GSLA based on their genetic and phenotypic relatedness (Salvetti et al., 2018).

Our pipeline is based on the mapping coverage which is thought to have a positive correlation with ANI. As expected, the relationship in most species showed a positive correlation, but such species including *E. faecalis* and *P. pentosaceus* had no correlation. This result may indicate a limitation of the ANI, as it only uses sequences with the best match in BLASTn after trimming the overall sequence to 1,020 bp (Arahal, 2014). Furthermore, cases, where the species classification was unclear based on the ANI, confirmed that ANI should be modified based on coverage or that a new method should be developed to address this problem (Rosselló-Móra and Amann, 2015; Varghese et al., 2015).

The classification programs used in this study required filtering of several FPs. Such filtering was easy when analyzing a single-species sample, but when multiple species were mixed, different filtering criteria were needed for accurate detection. That is, if information about the sample is not known, or if only a small amount of GSLA is present in the sample, the filtering value must be set blindly such that false detection cannot be controlled. This may lead to problems such as unresolved labeling errors.

As our pipeline involved the use of all reads mapped to the whole genome, the results of proportion analysis showed high consistency. Other classifications based on a specific sequence region of interest, such as those using *k*-mer value, had high variance values of two to three, showing that they are common for proportion analysis. Whole genomes were used to obtain more reliable results, which could be compared with identification data obtained using only 16S rRNA, as well as in the cases described above. For classification at the species level, it is difficult to obtain sufficient resolution with current sequencing technologies. Moreover, to conduct proportion analysis, a case-control study is the most commonly used method; furthermore, this method does not show errors when the amount of each species changes. However, targeting the 16S rRNA to determine the relative ratios of species is problematic because of differing numbers of 16S rRNA genes among species of microbes and the variation in copy numbers within species (Klappenbach et al., 2001).

In conclusion, we have shown that a pipeline using coverage was better in terms of coverage accuracy than other classification schemes. Constructing the reference dataset from representative strains was effective and allowed the pipeline to run with a reduced computational load. The reliable results obtained by our pipeline, with respect to GSLA detection (and proportions thereof) in probiotic products are expected to improve the quality of probiotics and associated safety management practices. Furthermore, although the microbes detected were limited to GSLA in this study, our pipeline can be extended to other microbes in the soil environment, viruses, and other microbial groups of interest.

## Author Contributions

WK designed the experiments, interpreted the data, and supervised the study. Funding, computing resources, and server time were granted by SC and HBK. DS performed the bioinformatic analyses, interpreted the data, and drafted the manuscript. HKK performed the experiments. S-YK provided the sequencing data for probiotic products and contributed to the discussion of the results. H-SK, SHK, WL, and SP provided critical comments and helped to direct the study. SYJ contributed to the revision and editing of the manuscript. All authors reviewed the manuscript.

## Conflict of Interest Statement

DS, SYJ, HKK, HBK, SC, and WK were employed by company C&K Genomics. S-YK was employed by company CTCBIO, Inc. The remaining authors declare that the research was conducted in the absence of any commercial or financial relationships that could be construed as a potential conflict of interest.

## Footnotes

1 https://www.gminsights.com/industry-analysis/probiotics-market

2 http://www.ncbi.nlm.nih.gov/

3 http://www.ncbi.nlm.nih.gov/SRA

4 https://github.com/asleofn/APD

5 https://drive.google.com/drive/folders/1fOakwxOp7QbxQooi8pHYjfrKIbxPuryl
