# Supplementary material for: Accurate and Strict Identification of Probiotic Species Based on Coverage of Whole-Metagenome Shotgun Sequencing Data
Source: Front Microbiol. 2019 Aug 7;10:1683. doi: 10.3389/fmicb.2019.01683 (PMC6693478; doi:10.3389/fmicb.2019.01683)
Supplement: Supplementary file 1 [file Data_Sheet_1.ZIP › Supplementary_Material.docx]

Supplementary Material

Accurate and Strict Identification of Probiotic Species Based on Coverage of Whole-metagenome Shotgun Sequencing Data

Donghyeok Seol, So Yun Jhang, Hyaekang Kim, Se-young Kim, HyoSun Kwak, Soon-Han Kim, Woojung Lee, Sewook Park, Heebal Kim, Seoae Cho and Woori Kwak*

*** Correspondence:** Woori Kwak: asleo@cnkgenomics.com

# Supplementary Figures and Tables

## Supplementary Figures

##
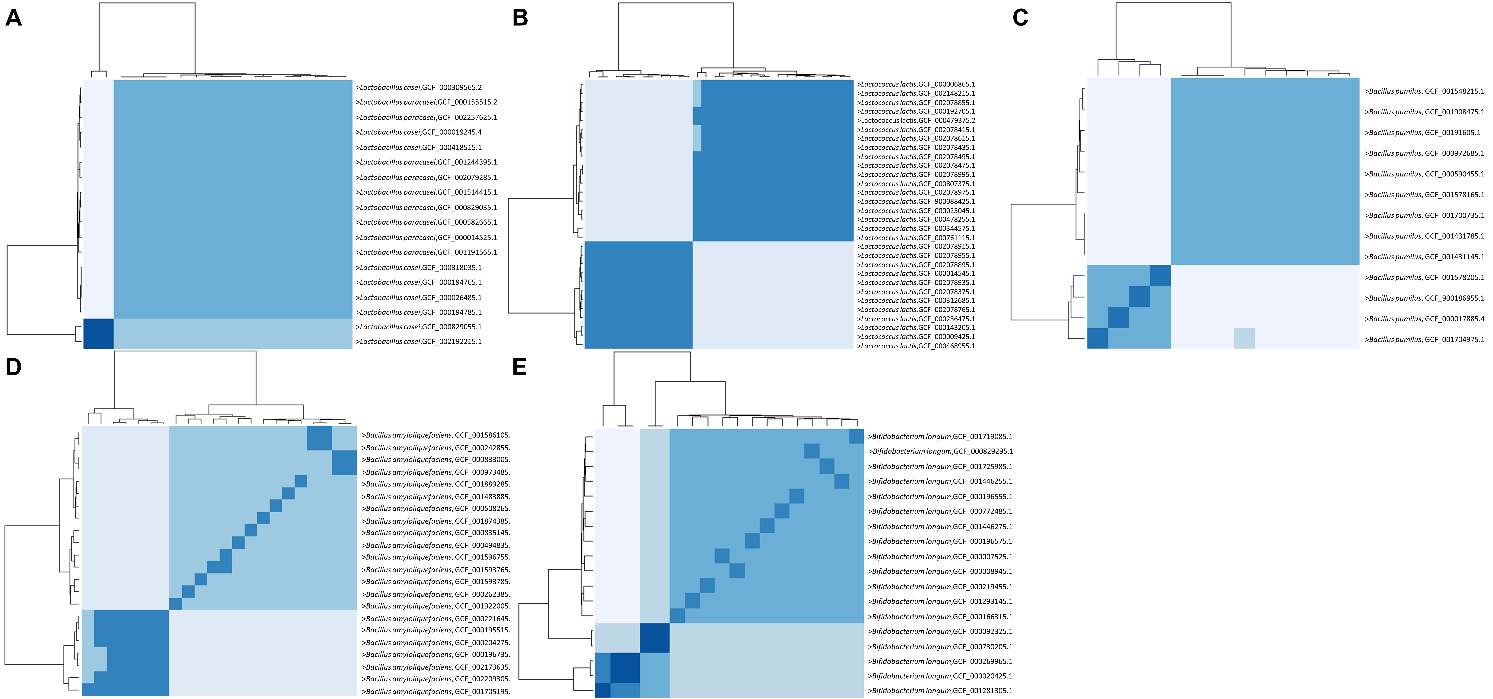


**Supplementary Figure 1.** Heatmap based on ANI value. Members of the same species were not always grouped together based on ANI. (A) shows *L. paracasei* + *L. casei* which were not distinguished by the ANI criterion, (B) shows *L. lactis*, (C) shows *B. pumilus*, (D) shows *B. amyloliquefaciens*, and (E) shows *B. longum*.


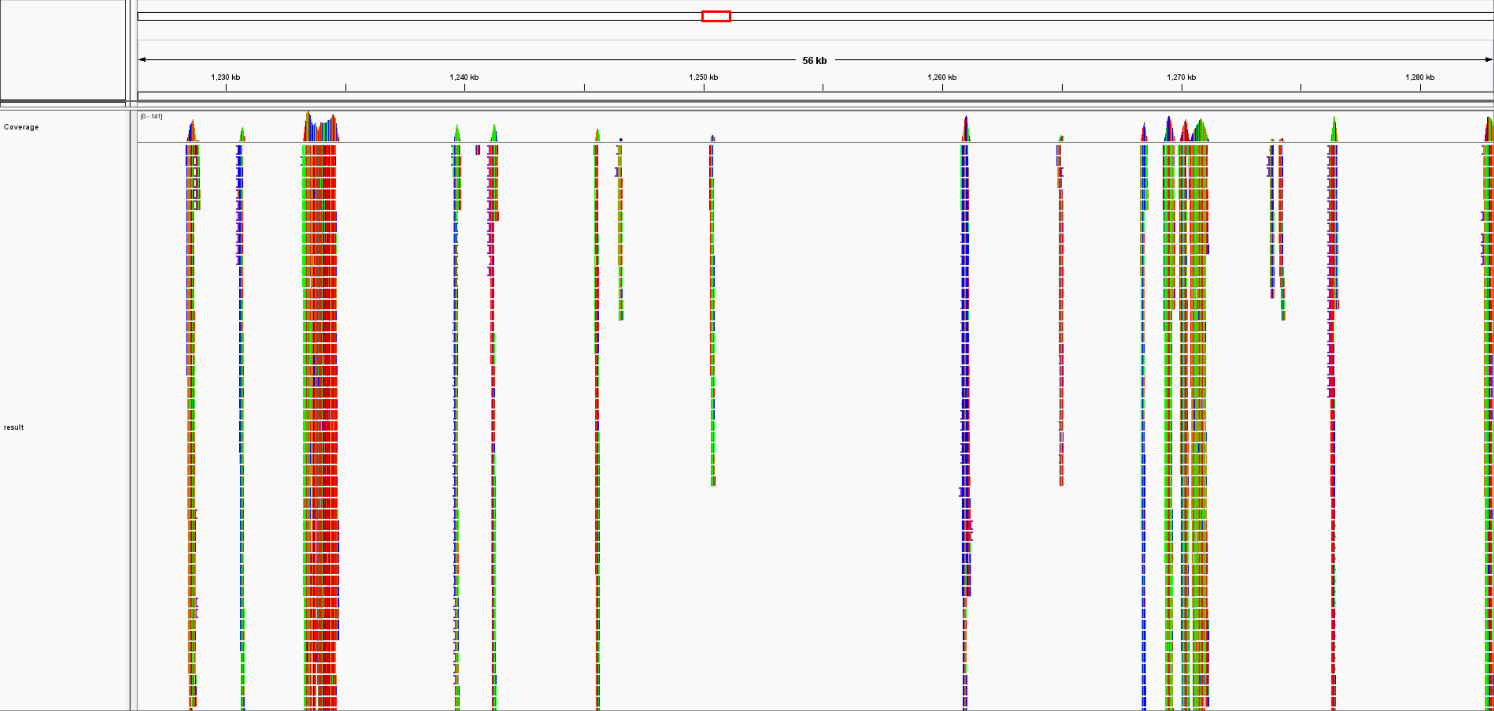


**Supplementary Figure 2.** Integrative Genomics Viewer image of *L. casei* in different group*.* When strain GCF_000019245.4 was aligned to strain GCF_000829055.1, the reads did not coverage fully, but only partially.


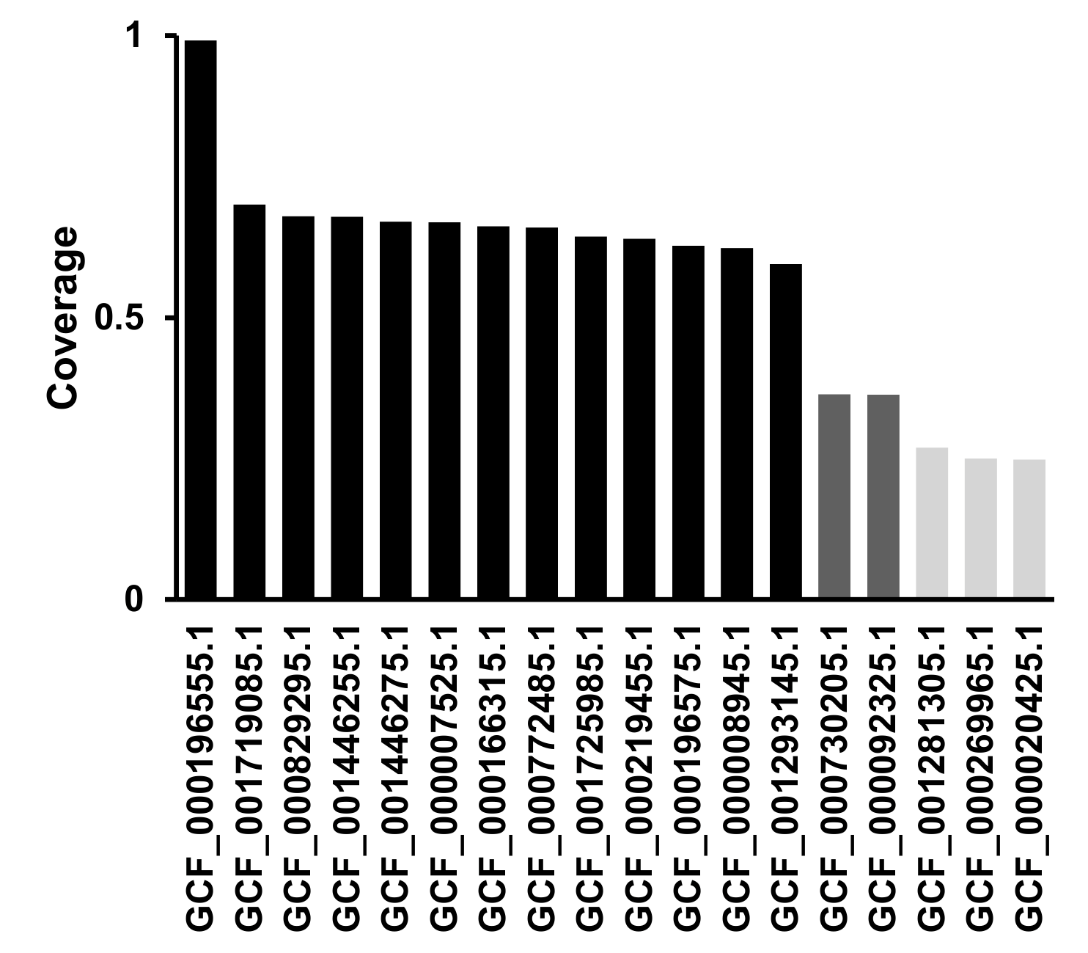


**Supplementary Figure 3.** Results when single isolated *B. longum* reads were aligned to the reference containing all *B. longum* strains. Results were divided into three groups based on coverage. Each group represents one subspecies of *B. longum*.


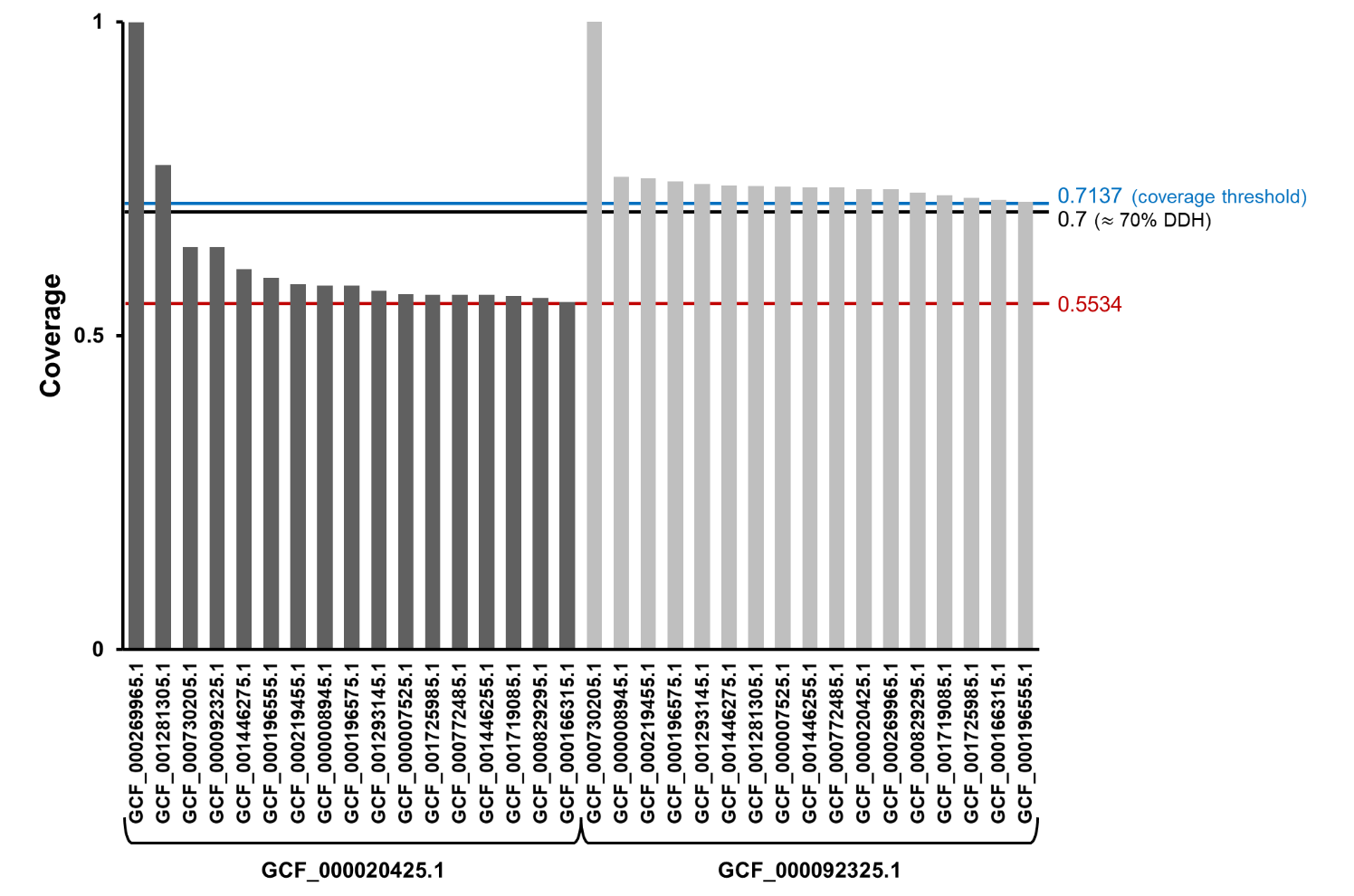


**Supplementary Figure 4.** Effect of representative selection on minimum coverage values. The values of one-to-one pairwise coverage for 18 strains of *B. longum* are shown with two strains, GCF_000092325.1 and GCF_000020425.1, used as the reference genome. The highest minimum coverage value among the 18 strains was obtained when strain GCF_000092325.1 was used as the reference, as shown by the blue line: 0.7137 coverage. When strain GCF_000020425.1 was used as the reference genome, the minimum coverage value was lowest for strain GCF_000166315.1, as shown by the red line: 0.5534 coverage. The black line, 0.7, indicates the DDH standard used for experimental identification.


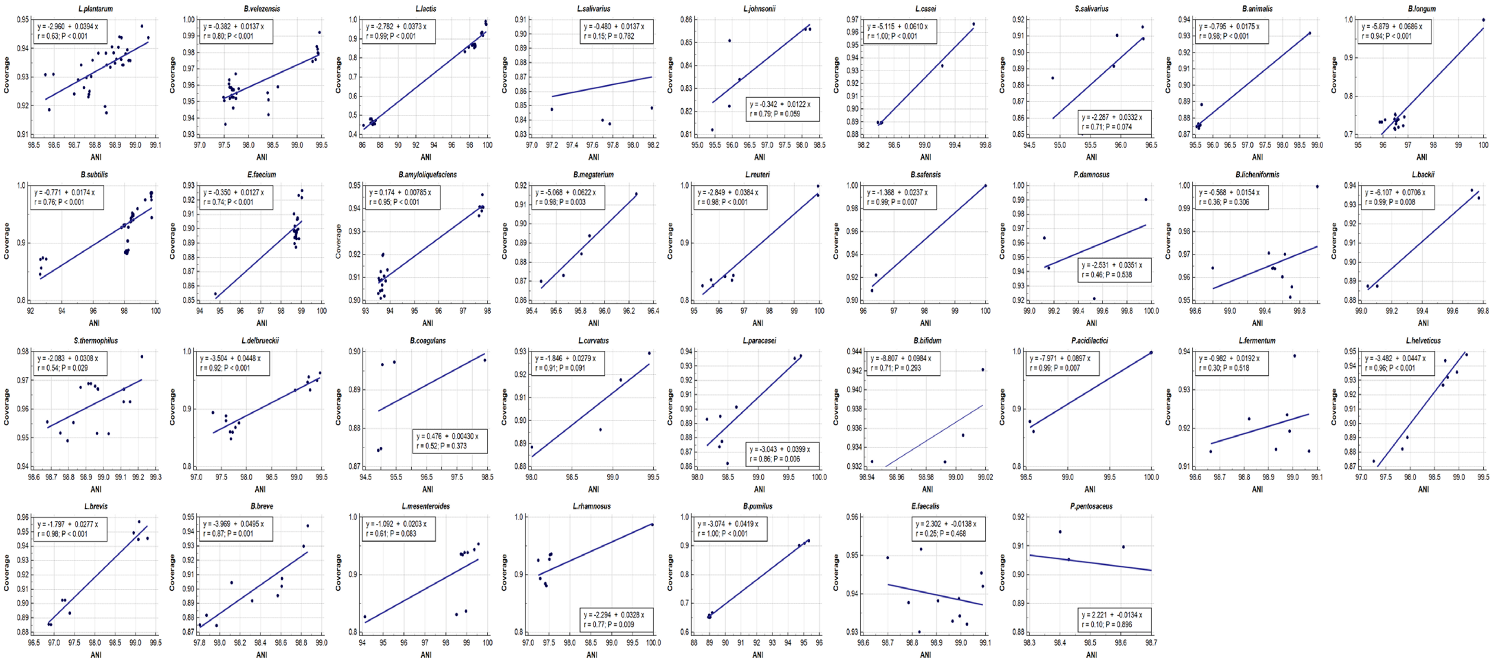


**Supplementary Figure 5.** The relationship between ANI and coverage*.* Each value indicates the relationship when the representative strain of a species was used as the reference sequence. GSLA species with more than five strains in the database are shown.


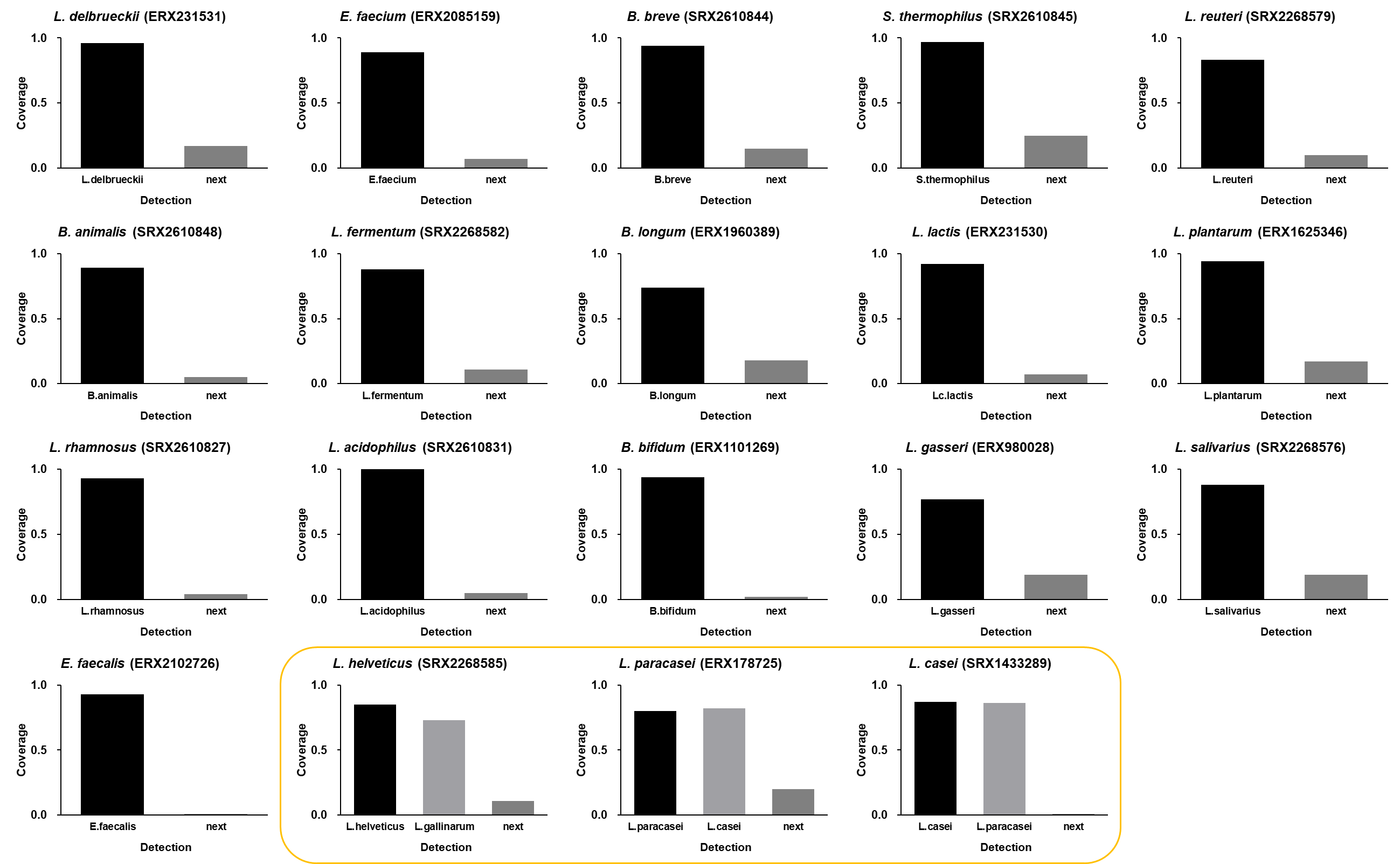


**Supplementary Figure 6.** Application to single isolated genome data from SRA. Three species were detected in the yellow square (*L. helveticus* (SRX2268585), *L. paracasei* (ERX178725) and *L. casei* (SRX1433289)) based on 0.7137 coverage. ‘next’ indicates the next highest coverage value when reads were aligned.

## Supplementary Tables

We uploaded supplementary tables as an additional excel file.
