# Supplementary figures and images for: Accurate and Strict Identification of Probiotic Species Based on Coverage of Whole-Metagenome Shotgun Sequencing Data
Source: Front Microbiol. 2019 Aug 7;10:1683. doi: 10.3389/fmicb.2019.01683 (PMC6693478; doi:10.3389/fmicb.2019.01683)

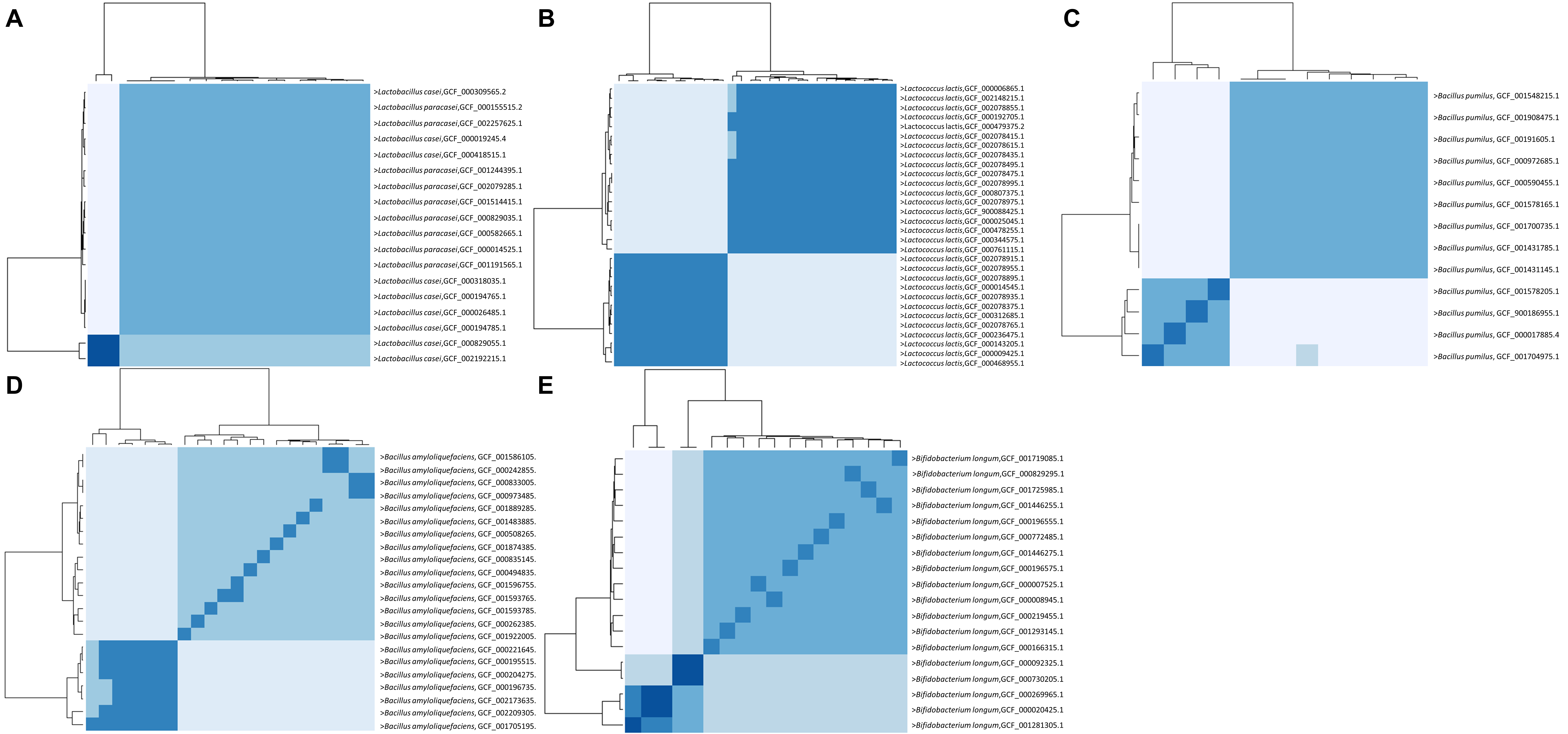

Supplement: Supplementary file 1 [file Data_Sheet_1.ZIP › Supplementary_Figure_1.tif]

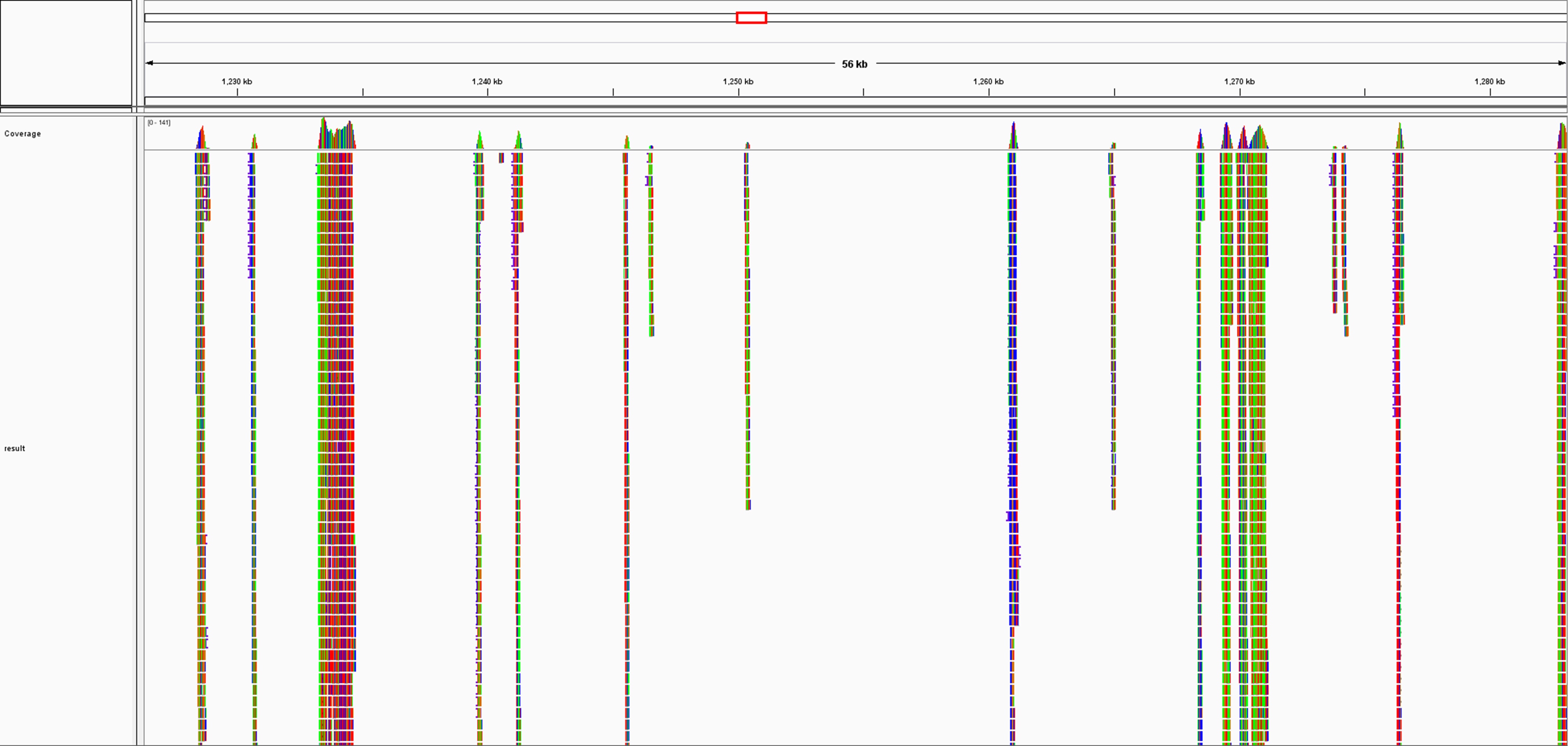

Supplement: Supplementary file 1 [file Data_Sheet_1.ZIP › Supplementary_Figure_2.tif]

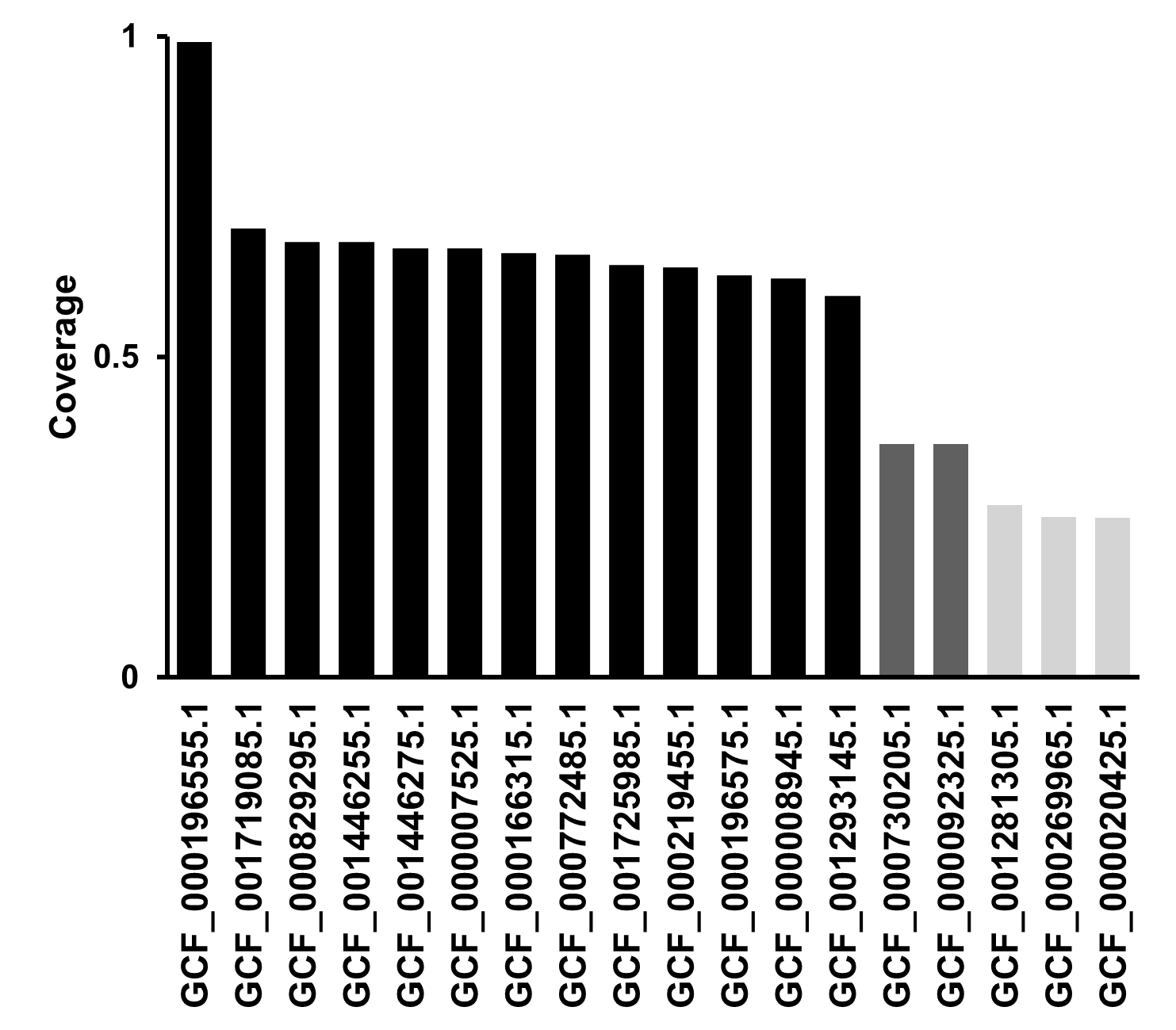

Supplement: Supplementary file 1 [file Data_Sheet_1.ZIP › Supplementary_Figure_3.tif]

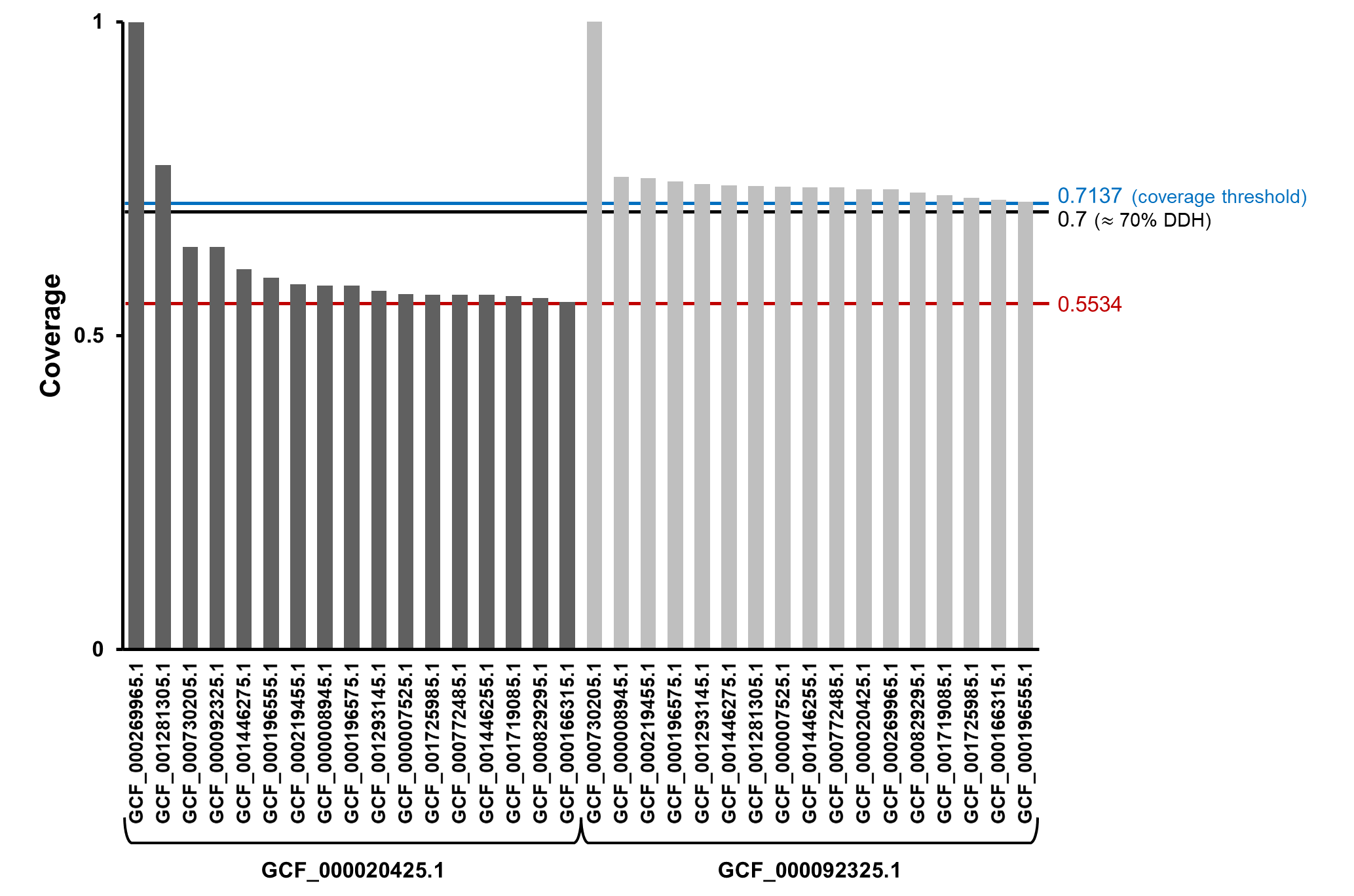

Supplement: Supplementary file 1 [file Data_Sheet_1.ZIP › Supplementary_Figure_4.tif]

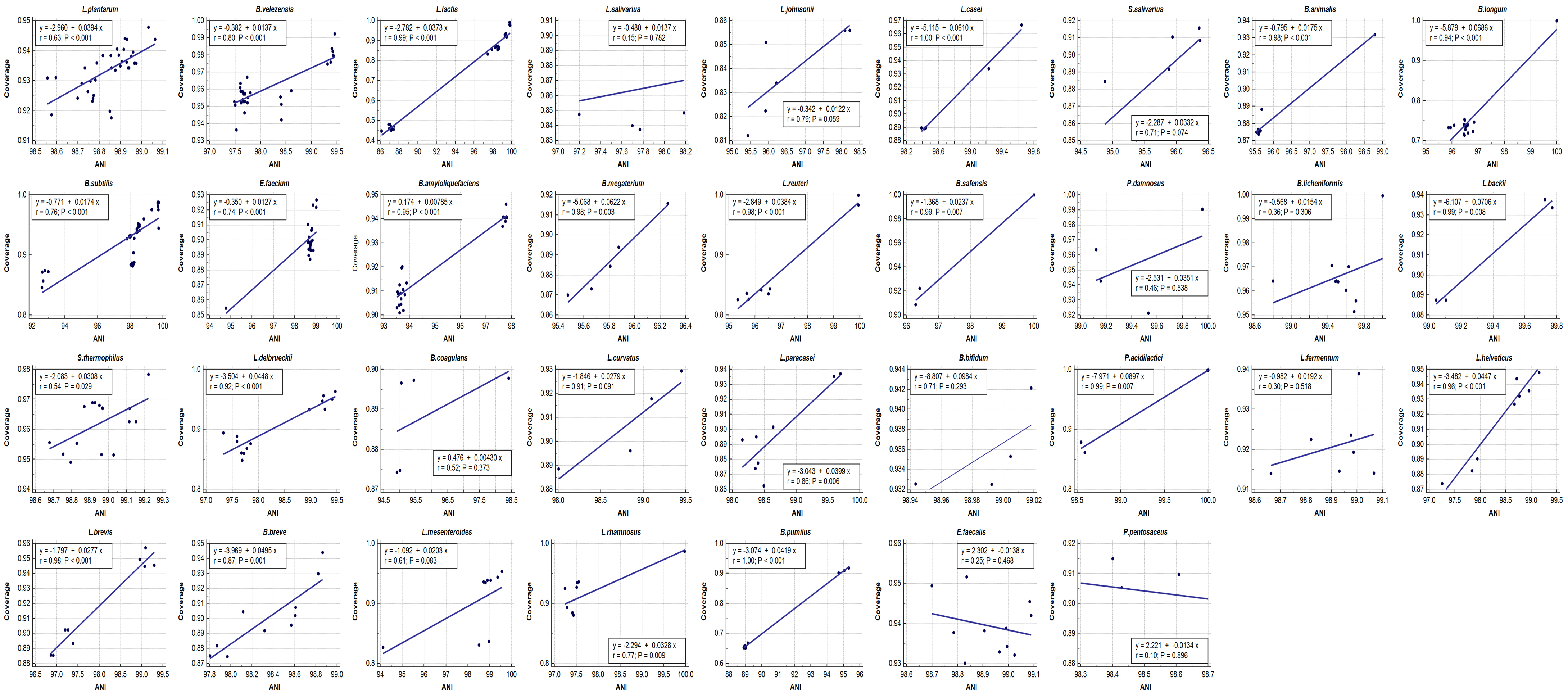

Supplement: Supplementary file 1 [file Data_Sheet_1.ZIP › Supplementary_Figure_5.tif]

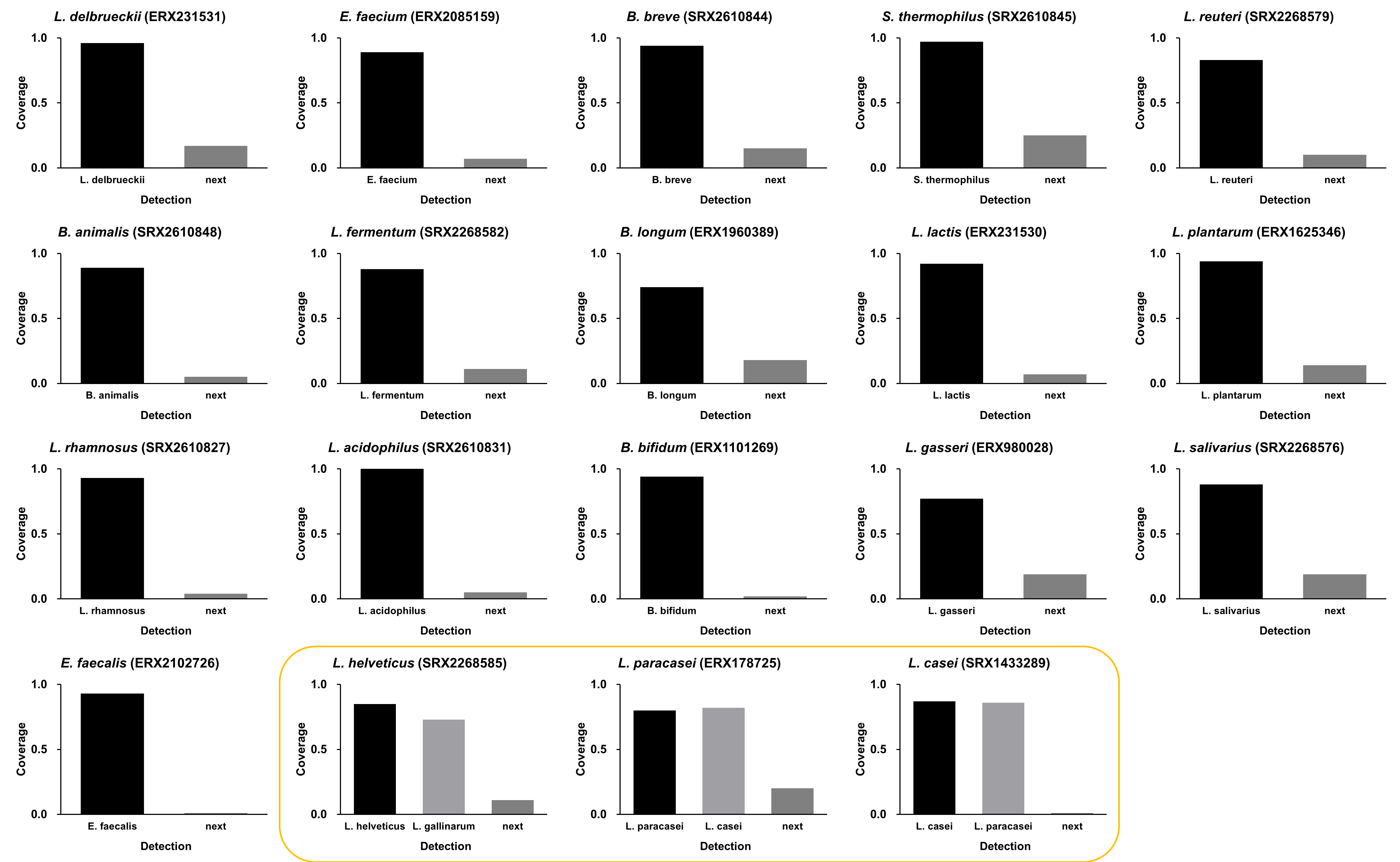

Supplement: Supplementary file 1 [file Data_Sheet_1.ZIP › Supplementary_Figure_6.tif]
